# Supplementary material for: Investigating the Prognostic Relevance of Tumor Immune Microenvironment and Immune Gene Assembly in Breast Carcinoma Subtypes
Source: Cancers (Basel). 2022 Apr 12;14(8):1942. doi: 10.3390/cancers14081942 (PMC9031175; doi:10.3390/cancers14081942)
Supplement: Supplementary file 1 [file cancers-14-01942-s001.zip › Supplementary_Material.pdf]

SUPPLEMENTARY

Investigating the prognostic relevance of tumor immune microenvironment and immune gene assembly in breast carcinoma subtypes

Supplementary Figures:

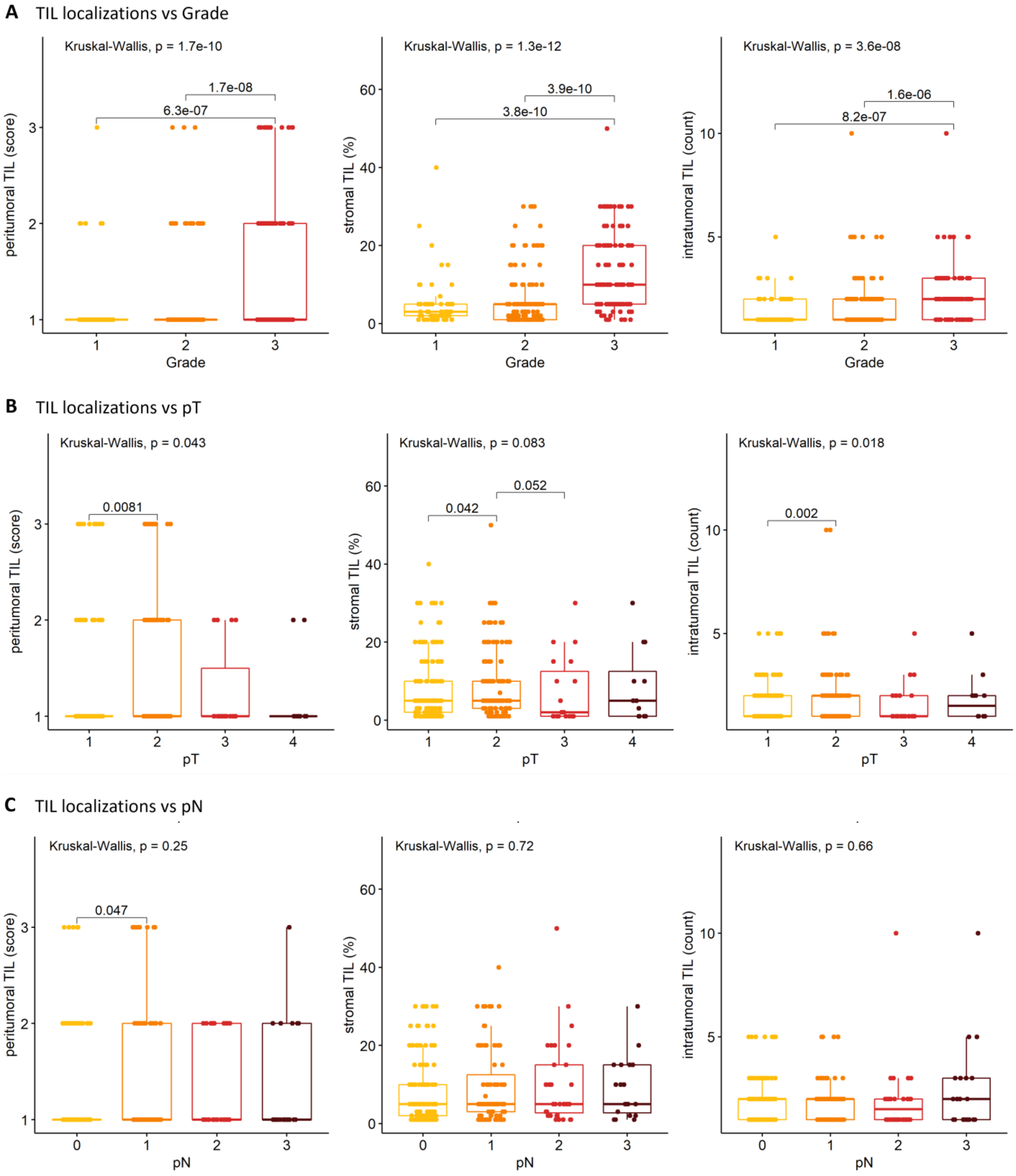

Supplementary Figure S1. The distribution of TIL categories across clinicopathological data. A, The distribution of grade (n = 309) in the different TIL localization categories. **B,** The distribution of pT status (n = 309) in the different TIL localization categories. **C,** The distribution of pN status (n = 276) in the different TIL localization categories. Global Kruskal-Wallis and Wilcoxon-tests were performed and only *p*-values < 0.100 are indicated.

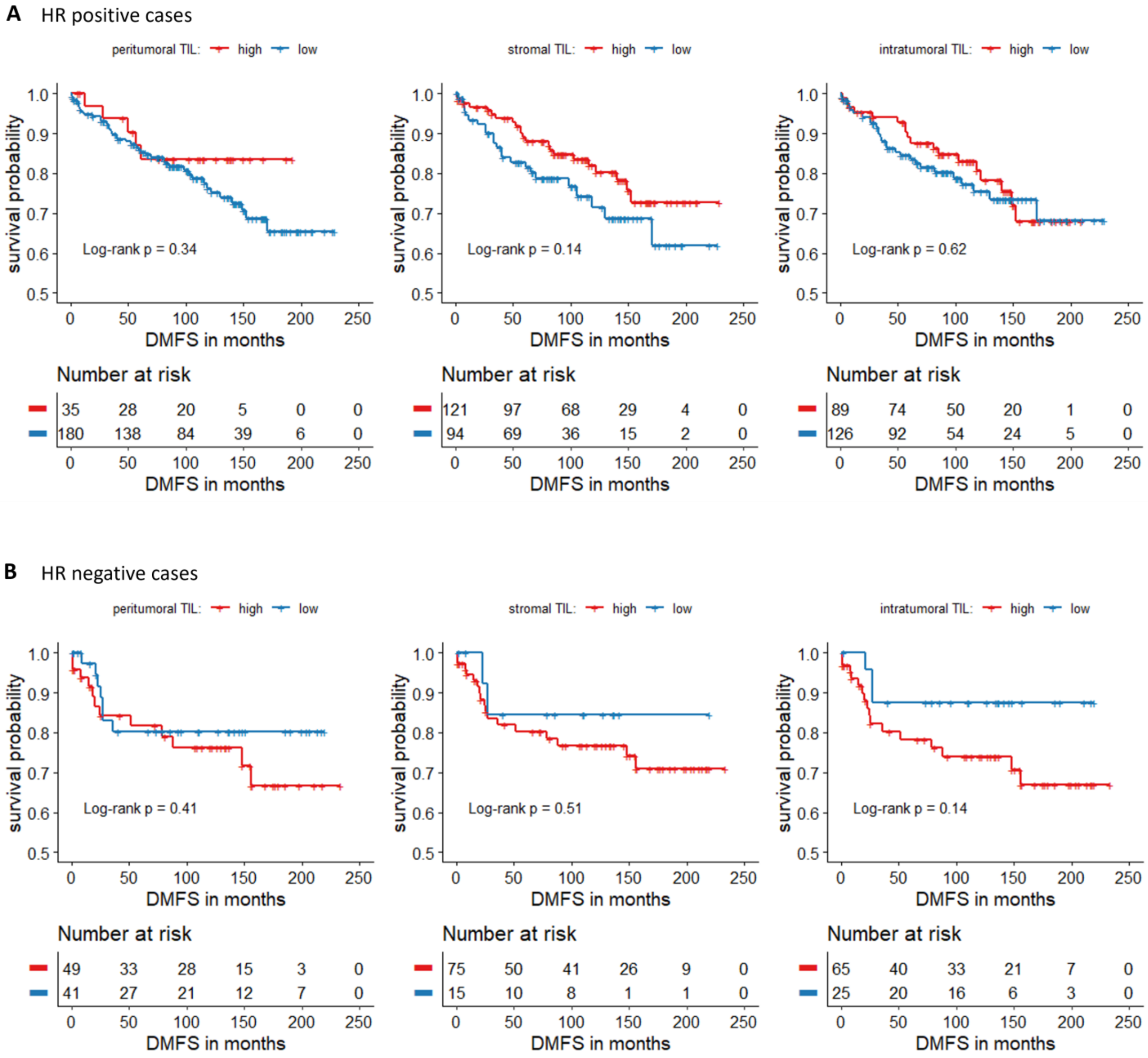

Supplementary Figure S2. Kaplan-Meier curves of HR positive and HR negative patient groups stratified based on high and low pTIL, sTIL, and iTIL. A, Kaplan-Meier curves of HR positive patients with high and low pTIL, sTIL and iTIL (n = 215). **B,** Kaplan-Meier curves of HR negative patients with high and low pTIL, sTIL and iTIL (n = 90).

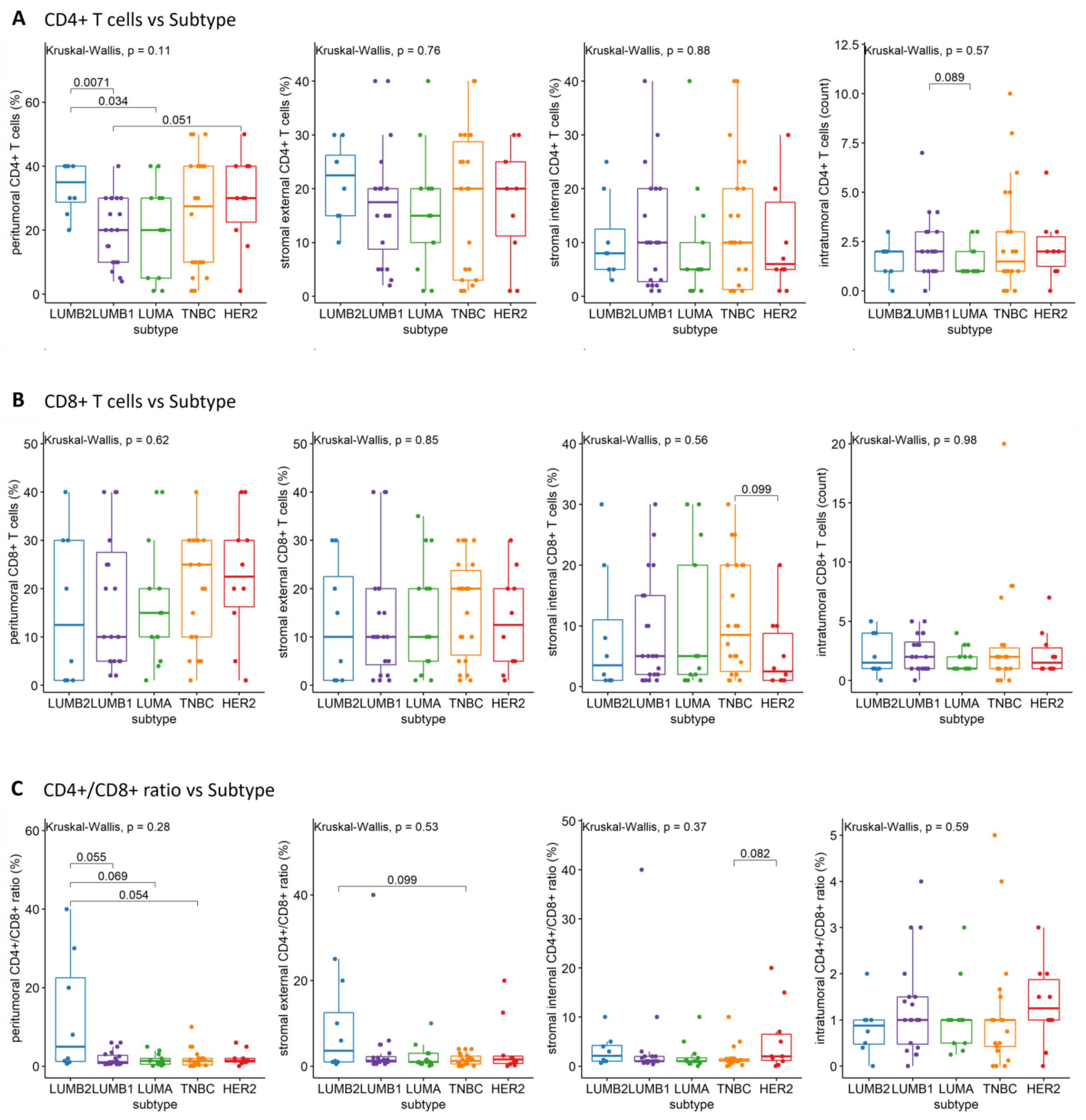

Supplementary Figure S3. Distribution of CD4$^+$, CD8$^+$ T cells as well as CD4$^+$/CD8$^+$ ratio in breast carcinoma subtypes. A, Association between CD4$^+$ T cell amounts at different localizations and subtype (number of observations from left to right: n = 72, n = 73, n = 73 and n = 73). **B,** Association between CD8$^+$ T cell amounts at different localizations and subtype (number of observations from left to right: n = 72, n = 73, n = 73 and n = 73). **C,** Association between CD4$^+$/CD8$^+$ ratio at different localizations and subtype (number of observations from left to right: n = 72, n = 73, n = 73 and n = 72). Global Kruskal-Wallis and Wilcoxon-tests were performed. Pairwise test results are shown only when *p*-values are less than 0.100.

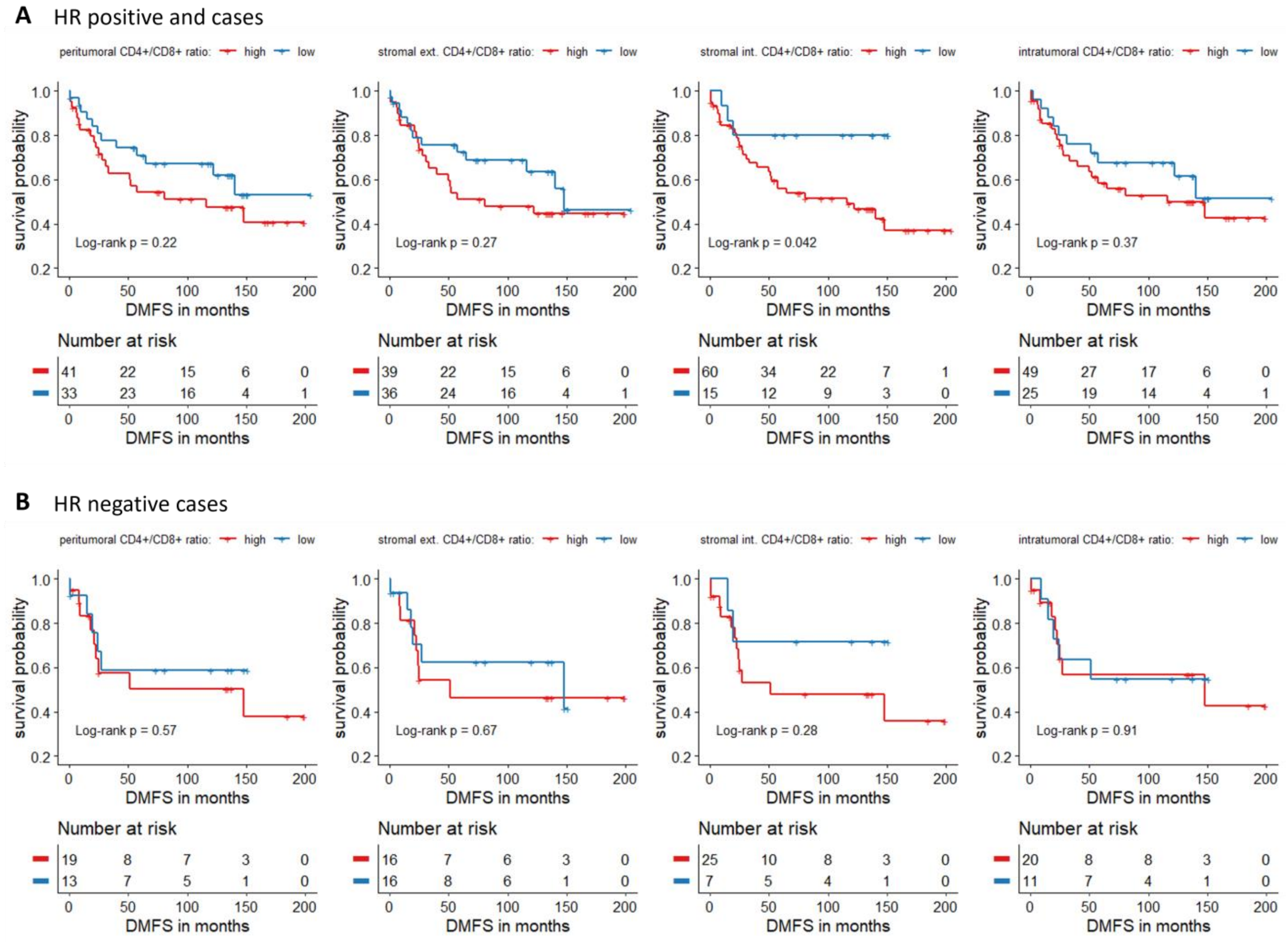

Supplementary Figure S4. Relationship between CD4$^+$/CD8$^+$ ratio at different localizations and DMFS. A, Kaplan-Meier curves of high and low CD4$^+$/CD8$^+$ ratio at different localizations (number of observations from left to right: n = 74, n = 75, n = 75 and n = 74). **B,** Kaplan-Meier curves of high and low CD4$^+$/CD8$^+$ ratio at different localizations, restricting the dataset only to HR negative cases (number of observations from left to right: n = 32, n = 32, n = 32 and n = 31).

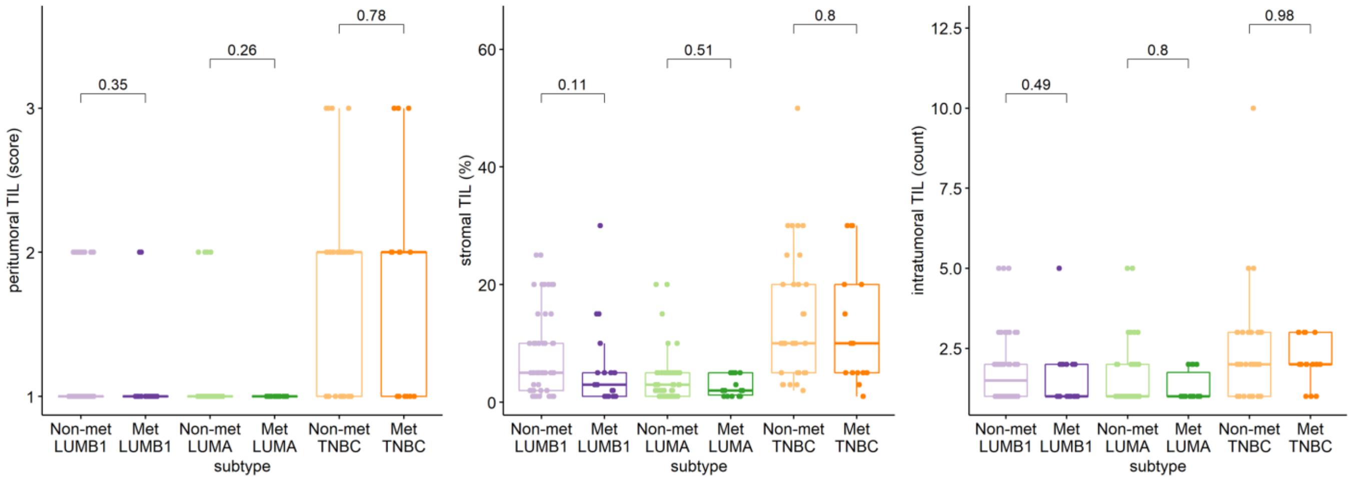

Supplementary Figure S5. Peritumoral, stromal and intratumoral TIL distribution in metastatic vs. non-metastatic cases in the three breast carcinoma subtypes investigated on HE stained slides (n = 203). Pairwise Wilcoxon-tests were performed, and all *p*-values are indicated.

Supplementary Tables:

Table S1. The CD4+ and CD8+ T cell distributions in the different tumor compartments.

Cases	Sub-type	CD4+ T cells (%)/ peri-tumoral	CD8+ T cells (%)/ peri-tumoral	CD4+ T cells (%)/ stromal external	CD8+ T cells (%)/ stromal external	CD4+ T cells (%)/ stromal internal	CD8+ T cells (%)/ stromal internal	CD4+ T cells (count)/ intra-tumoral	CD8+ T cells (count)/ intra-tumoral
**1**	LUMB1	25	10	15	10	10	5	2	2
**2**	LUMA	20	15	10	1	10	1	3	1
**3**	LUMB1	7	2	5	1	1	1	1	1
**4**	LUMB2	20	1	10	1	10	1	2	1
**5**	TNBC	40	30	20	20	15	10	3	2
**6**	TNBC	30	30	30	20	40	25	3	7
**7**	LUMA	1	5	1	10	1	30	1	1
**8**	NA	30	30	10	15	5	10	2	2
**9**	TNBC	30	10	20	5	20	5	5	3
**10**	LUMB1	30	5	30	5	20	2	3	1
**11**	TNBC	10	30	10	15	5	5	1	1
**12**	LUMA	1	10	1	2	1	2	1	2
**13**	HER2	40	30	30	25	30	2	2	2
**14**	TNBC	1	10	1	20	1	5	1	2
**15**	HER2	30	20	10	5	5	1	1	1
**16**	HER2	30	5	20	1	5	3	2	1
**17**	LUMB1	30	5	25	20	20	20	3	1
**18**	LUMB1	30	10	20	10	15	5	7	5
**19**	LUMB2	40	30	30	30	20	30	2	5
**20**	LUMB2	40	5	30	5	3	1	1	1
**21**	LUMA	20	40	10	30	15	25	1	2
**22**	LUMB1	NA	NA	40	1	40	1	2	1
**23**	LUMB1	30	20	15	15	20	20	1	4
**24**	TNBC	10	1	3	1	1	1	1	1
**25**	LUMB1	4	5	3	1	3	3	4	1
**26**	LUMB2	40	1	25	1	8	2	1	1
**27**	TNBC	50	10	25	10	10	1	5	1
**28**	LUMA	40	30	30	35	40	30	1	4
**29**	TNBC	40	30	30	20	25	20	8	2
**30**	LUMB2	40	20	15	30	8	8	2	2
**31**	TNBC	1	30	1	20	1	30	0	0
**32**	LUMB1	5	10	2	2	1	1	1	1
**33**	LUMB1	10	2	5	5	2	2	1	1
**34**	LUMB1	10	10	10	2	2	2	4	3
**35**	TNBC	10	5	5	2	2	2	1	1
**36**	TNBC	40	20	40	10	20	4	2	1
**37**	LUMA	5	10	20	20	5	2	2	2
**38**	TNBC	40	25	30	20	10	10	2	2
**39**	TNBC	10	30	5	30	10	15	1	3
**40**	HER2	40	20	25	2	20	1	3	1

**41**	TNBC	50	30	40	25	25	20	6	8
**42**	HER2	50	25	30	15	7	1	2	1
**43**	NA	40	30	25	15	20	20	5	5
**44**	TNBC	25	5	20	5	15	10	1	1
**45**	LUMB1	10	5	15	10	5	5	2	1
**46**	LUMB1	20	30	20	20	3	5	3	3
**47**	LUMA	20	4	15	5	5	5	2	1
**48**	TNBC	1	15	1	10	1	2	0	0
**49**	LUMA	30	15	20	20	5	3	1	3
**50**	LUMB1	15	20	15	20	10	15	1	3
**51**	LUMA	20	20	15	5	5	5	1	1
**52**	HER2	40	40	20	20	10	5	3	2
**53**	HER2	15	1	1	5	1	1	1	1
**54**	HER2	20	15	25	10	20	10	6	4
**55**	LUMB1	40	40	40	40	30	30	2	5
**56**	LUMA	5	1	5	1	5	1	1	1
**57**	HER2	30	40	15	30	5	20	2	7
**58**	LUMB1	30	30	20	15	10	10	2	4
**59**	TNBC	5	20	3	30	5	20	1	8
**60**	LUMB2	25	40	25	20	25	20	2	4
**61**	TNBC	40	40	25	30	40	25	10	20
**62**	TNBC	40	30	30	30	30	20	2	2
**63**	LUMA	40	40	40	30	20	20	3	3
**64**	TNBC	10	5	2	1	1	1	0	0
**65**	TNBC	50	30	25	25	10	7	2	2
**66**	TNBC	5	25	1	2	1	1	1	1
**67**	LUMB1	15	25	5	10	2	1	0	0
**68**	LUMB1	20	40	20	40	10	25	1	4
**69**	LUMA	30	20	20	20	1	1	1	1
**70**	LUMB2	30	1	20	1	5	1	0	0
**71**	LUMA	30	10	15	10	5	5	1	1
**72**	LUMB2	30	30	15	15	5	5	3	4
**73**	LUMB1	25	25	20	10	10	10	3	2
**74**	LUMB1	20	40	20	40	25	15	2	2
**75**	HER2	1	30	1	20	1	10	0	3
